# Association between Toenail Mercury and Metabolic Syndrome Is Modified by Selenium

**DOI:** 10.3390/nu8070424

**Published:** 2016-07-12

**Authors:** Kyong Park, Eunmin Seo

**Affiliations:** Department of Food and Nutrition, Yeungnam University, Gyeongsan 38541, Gyeongbuk, Korea; daworu@ynu.ac.kr

**Keywords:** toenail mercury, metabolic syndrome, effect-modification, selenium, Asian

## Abstract

Background: Although Asian populations consume relatively large amounts of fish and seafood and have a high prevalence of metabolic diseases, few studies have investigated the association between chronic mercury exposure and metabolic syndrome and its effect modification by selenium. Methods: We analyzed baseline data from the Trace Element Study of Korean Adults in the Yeungnam area. Participants included 232 men and 269 women, aged 35 years or older, who had complete data regarding demographic, lifestyle, diet, toenail mercury and selenium levels, and health. Toenail mercury and selenium concentrations were measured using instrumental neutron-activation analysis. The metabolic biomarker levels were obtained through biannual medical checkups. Results: Higher toenail mercury levels were associated with habitual consumption of whale and shark meats, older age, obesity, smoking, alcohol drinking, and higher household income. Multivariable analysis showed a positive association between toenail mercury exposure and metabolic syndrome. In addition, this association was significantly stronger at lower selenium levels and was weaker at higher selenium levels. Conclusion: The possible harmful effects of mercury on metabolic syndrome may be attenuated by high levels of selenium. Future studies are needed to suggest optimal dietary guidelines regarding fish and selenium intakes, particularly for Asians with high levels of fish intake.

## 1. Introduction

Metabolic syndrome (MetS) is an increasing public health burden, leading to increased risk of other metabolic diseases, including diabetes and cardiovascular disease [1,2,3]. In particular, Asian populations have relatively lower body mass index (BMI) and obesity rates than Western populations, but the prevalence of MetS has been increasing [4,5]. Considering that Asian society has been aging, it is anticipated that this increasing trend will continue; thus, it is critical to identify risk factors associated with MetS in Asian populations.

Many Asians frequently consume fish and seafood in larger amounts than do Western populations [6,7,8]. Fish intake has been considered to have beneficial effects on prevention of chronic diseases [9]. However, some fish species contain methylmercury, which may be toxic for metabolic health conditions [9,10]. Higher fish consumption may lead to accumulation of methylmercury in the human body [11,12,13,14,15]. Cumulative evidence has shown that chronic mercury exposure may induce oxidative stress [16,17,18] and inflammation [19], eventually leading to increased risk of insulin resistance and metabolic syndrome [20]. In addition, fish also contains selenium, a trace nutrient that may counter the toxic effects of methylmercury [21].

A few recent studies demonstrated a positive association between chronic mercury exposure and MetS, mainly in the Korean population, but the results were inconclusive. In several Korean cross-sectional studies, individuals with high concentrations of mercury in the blood or hair were more likely to have MetS [22,23], while studies using the Korean National Health and Nutrition Examination Survey (KNHANES) data showed discrepant results [24,25,26,27]. However, all prior studies used blood or hair biomarkers. Blood mercury concentration may represent short-term exposure to mercury, and the hair mercury concentration may be limited by possible contamination from the use of hair products. In addition, it is not clear whether the association between mercury levels and MetS is modified by selenium.

Accordingly, we conducted a cross-sectional study to examine the association between toenail mercury levels and MetS and to determine whether selenium in toenails would modify this association.

## 2. Materials and Methods

### 2.1. Population and Design

The Trace Element Study of Korean Adults in the Yeungnam area (SELEN) is a prospective cohort study of community-dwelling healthy individuals aged 35 years or older in southeastern South Korea (Yeungnam area). At baseline, 740 individuals were recruited through advertisements. Participants completed questionnaires concerning demographic, lifestyle, and dietary information, and provided toenail clippings between December 2012 and December 2013. For this analysis, we included 232 men and 269 women with complete data regarding demographic, lifestyle, and health examination, as well as toenail mercury and selenium levels. Participants included in this study were three years younger and had lower rates of current smoking than those excluded, but most other characteristics, such as sex, BMI, education income, physical activity, alcohol consumption, residence area, dietary supplement use, or family history of chronic diseases, did not differ. All participants provided written consent, and the study was approved by the Institutional Review Board of the Yeungnam University Medical Centre (YUH-12-0468-O94).

### 2.2. Assessment of Toenail Mercury and Selenium Levels

Mercury and selenium levels in toenail clippings reflect long-term dietary exposure of up to one year [28,29,30,31,32]. We collected toenail clippings from all 10 toes of each participant and inspected to eliminate contaminated samples such as manicured nails or nails dyed with garden balsam. Inspected and clean toenail clippings were stored in a paper envelope at room temperature and shipped to the University of Missouri Research Reactor (Columbia, MO, USA) via express mail. Before the procedure, toenail clippings were washed in a sonicator with deionized water. Toenail mercury and selenium levels were quantified using neutron activation analysis, a nuclear process for evaluating the levels of trace elements at the University of Missouri Research Reactor. Details of the analytic methods and validation of the measures have been previously published [28,33,34,35].

### 2.3. Assessment of Dietary and Lifestyle Information

Fish and seafood intake levels were quantified using 23 items of a semi-quantitative food frequency questionnaire (FFQ), including consumption of whale and shark. Daily consumption levels were calculated as grams; the frequency intake of food item per day was multiplied by the reported portion size. In the FFQ, a standardized portion size (reference size) was provided with three answer options: small (0.5 times reference), medium (reference), and large (1.5 times of reference).

Education level was classified into two groups—high school graduation or less and college graduation or more. Income level was classified into five groups based on monthly household income. Participants self-reported whether they were current smokers, former smokers, or had never smoked. Since the frequency of non-alcohol drinkers was high, alcohol consumption status was categorized as drinker or non-drinker. Physical activity levels were determined using metabolic equivalents (METs) h/week, calculating time participants had spent in vigorous and moderate activities and walking during the past week with weighted frequency and duration of activities [36].

### 2.4. Assessment of Anthropometry and Metabolic Biomarkers

Participant’s health information, including anthropometric and metabolic biomarker measurements, were collected through biannual medical checkups conducted by the Korea National Health Insurance Service [37]. At least six months after the completion of the baseline survey and toenail sample collection, participants had the medical-checkups and sent a copy of their medical checkup results to the SELEN investigators. Height, weight, and waist circumference were recorded by a trained nurse at each clinic, and BMI was calculated as weight in kilograms divided by the square of height in meters. Before the medical checkups, participants fasted for at least 12 h, and serum concentrations of triglycerides, cholesterol, and glucose were measured at qualified clinical laboratories with standard quality assurance and control protocols in place [37].

MetS was defined based on the National Cholesterol Education Program Adult Treatment Panel III (NCEP APT III) criteria [38] with a modified cutoff of waist circumference for an Asian population [39,40]. A MetS diagnosis required at least three of the following: (1) a waist circumference ≥90 cm in men and ≥80 cm in women; (2) fasting glucose ≥100 mg/dL; (3) fasting triglycerides ≥150 mg/dL; (4) HDL cholesterol <40 mg/dL in men and <50 mg/dL in women; and (5) systolic blood pressure ≥130 mmHg and/or diastolic blood pressure ≥85 mmHg.

### 2.5. Statistical Analysis

Toenail mercury level was logarithmically transformed, given that its distribution was skewed to the right. To identify independent determinants of toenail mercury, demographic, lifestyle, and dietary variables were evaluated with log-transformed toenail mercury values in a generalized linear model. The age-adjusted mean levels of multiple metabolic biomarkers were compared between the sex-specific tertiles of toenail mercury, and tests for trends were conducted by using the tertile median value for toenail mercury in a generalized linear regression. Potential confounding variables were determined based on previous research results and preliminary analysis, and effect modification was tested for multiple demographic and lifestyle variables using multiplicative terms in the logistic regression and stratified analysis. We found that the association between toenail mercury and MetS was significantly altered by selenium levels, thus we reported the results stratified by selenium levels. Odds ratios and 95% confidence intervals (CI) of MetS were estimated using multivariable logistic regression, adjusting for potential confounding variables. SAS version 9.3 (Cary, NC, USA) was used for all analyses, and a critical value for *p* was set at α = 0.05, two-tailed.

## 3. Results

Characteristics of the study participants are presented in Table 1. The average age was 44.8 ± 0.24 years, and 46.5% were men. Approximately half the participants were overweight or obese. The proportion of current smokers was only 18.5%, whereas the proportion of current drinkers was 78.5%. Dietary supplement use was common, with approximately half the participants reporting using one or more multivitamins or similar dietary product. The mean levels of toenail mercury and selenium were 0.40 µg/g and 0.69 µg/g, respectively.

We examined demographic, lifestyle, and dietary factors to identify independent correlates of toenail mercury, adjusting for each of the factors in the multivariable linear regression model (Table 2). Age was an independent correlate of toenail mercury concentration, indicating that older participants had a higher toenail mercury concentration. Obesity and current smoking were all independently associated with higher concentrations of toenail mercury and demonstrated a dose-response relationship. Compared to underweight participants, the toenail mercury concentration was 24.6% higher in normal weight (*p* = 0.09), 30.5% higher in overweight (*p* = 0.04), and 45% higher in obese participants (*p* = 0.003). When compared to non-smokers, former and current smokers had 14.2% (*p* = 0.09) and 23.1% (*p* = 0.005) higher concentrations of toenail mercury, respectively. Alcohol consumption was also an independent correlate, as toenail mercury concentration was 14.8% higher in current alcohol drinkers than in non-drinkers (*p* = 0.01). Participants with higher income were more likely to have higher concentrations of toenail mercury, particularly for those with 5,000,000 Korean won or more of monthly household income (*p* < 0.05). In this study, toenail mercury concentration did not differ between levels of total fish intake, but did differ between intake levels of shark and whale meat—large marine species containing relatively high levels of methylmercury.

Age-adjusted anthropometry and metabolic biomarker levels were compared according to the sex-specific tertiles of toenail mercury shown in Table 3. Obesity markers (BMI and waist circumference), blood pressure (systolic and diastolic), fasting blood glucose, and triglyceride levels were all significantly associated with the toenail mercury concentration with a dose-response relationship (*p* for trend < 0.001). However, cholesterol (total, high-density lipoprotein, low-density lipoprotein) levels were not different between tertiles of toenail mercury.

Table 4 shows the odds ratio of MetS according to sex-specific tertiles of toenail mercury concentrations. In an age-adjusted model, the association between the toenail mercury concentration and MetS was not statistically significant (*p* for trend = 0.06). In a fully adjusted model, however, this association strengthened and became significant. Compared to participants in the lowest tertile of toenail mercury concentration, those in the highest tertile of toenail mercury concentration were 2.47 times more likely to have MetS (95% CI 1.01–6.08, *p* for trend = 0.03).

This positive association between toenail mercury and MetS remained significant and strengthened at below-median levels of participants’ toenail selenium (≤0.685 μg/g, Table 5); participants in the third tertile of toenail mercury concentration were 3.97 times more likely to have MetS (95% CI 1.15–13.76) compared to those in the first tertile (*p* for trend = 0.02). However, the association between toenail mercury and MetS was remarkably attenuated and became non-significant above median levels of participants’ toenail selenium (>0.685 μg/g), with odds ratios (95% CIs) of 0.95 (0.21–4.31) and 1.56 (0.39–6.20) in the second and third tertiles of toenail mercury, respectively (*p* for trend = 0.5).

## 4. Discussion

In this cross-sectional study, higher toenail mercury levels were associated with the prevalence of MetS, and the association between toenail mercury and MetS was influenced by selenium levels, indicating that this association was significantly stronger at lower selenium levels and weaker at higher selenium levels. The majority of study participants were residents in urban and rural southeastern areas (the Yeungnam area) in South Korea. Residents of the Yeungnam area have the highest blood mercury concentrations compared to those in other regions of South Korea [41], and they habitually consume whale and shark meat, which were revealed as independent correlates of toenail mercury concentration in this study. However, variations in fish and seafood consumption were not associated with the toenail mercury concentration of the study participants. In addition, older age, obesity, smoking, alcohol drinking, and higher household income were identified as independent correlates, after adjustment for diet. Higher concentrations of toenail mercury were associated with several metabolic risk factors, including higher systolic and diastolic blood pressure, fasting blood glucose, and triglyceride levels, as well as with MetS.

Although total fish intake was not a significant predictor of toenail mercury levels in the participants of this study, it is well known that higher fish consumption leads to accumulation of methylmercury in the human body. Participants in this study mainly resided in the Yeungnam area, where the residents have higher blood mercury levels than those residing in other regions of Korea because of traditional regional dietary culture (habitual consumption of shark and whale meat) [41]. Thus, when we included both variables of shark/whale meat and total fish intakes in the regression model simultaneously, variability in toenail mercury levels, as shown by the *R*^2^ value, may be explained by the greater shark/whale meat intake relative to the total fish intake. As a result, a variable of shark/whale meat intake remained as a strong significant predictor of toenail mercury levels, whereas total fish intake did not.

Previous studies regarding the association between mercury exposure and MetS are limited, and most were conducted in limited geographic areas with whole blood mercury used as the biomarker. Eom et al. [23] conducted a cross-sectional study with 2114 participants who were recruited by probability sampling methods covering all metropolitan, urban, and rural districts of South Korea; there was a significant positive association between mercury exposure and the prevalence of MetS, and this association remained significant after adjusting for age, sex, smoking, drinking alcohol, income, residence area, and seafood intake. Moon [26] confirmed this positive association using data from the 2009 to 2010 KNHANES, a nationally representative survey of the non-institutionalized Korean civilian population; mercury levels were associated with the prevalence of MetS in an adjusted model for age, sex, region, smoking, alcohol consumption, and regular exercise. This significant finding was also observed in a study by Chung et al. [27] using data from the 2010 to 2012 KNHANES for male Korean adults only. Thus, the present results are consistent with previous research indicating that people with higher mercury concentrations are more likely to have MetS.

The mechanism by which chronic mercury exposure is associated with MetS has not been fully elucidated, but is believed to involve an increase in oxidative stress, in which mercury may increase the production of free radicals [16,17,18] and disrupt activation of the antioxidant function of glutathione by binding to sulfhydryl groups [42], eventually leading to increased oxidative stress and an increased risk of MetS. In addition, cumulative evidence from experimental studies has shown that mercury may induce endoplasmic reticulum stress and inflammation [19], which is directly linked to obesity as well as insulin resistance and metabolic syndrome [20].

Interestingly, the association between toenail mercury and MetS is modified by levels of toenail selenium. Participants with chronic exposure to mercury were much more likely to have MetS at a lower selenium level, while this association became weaker and non-significant at a higher selenium level. Prior studies suggest that selenium deficiency may increase the risk of metabolic diseases such as diabetes [43] and diabetes complications [44]. Selenium is an essential nutrient for normal immune function and production of selenium-containing enzymes, many of which play a role in the body’s antioxidant defense system. In a randomized trial, researchers observed that pubic hair mercury concentrations of subjects with low serum levels of selenium were substantially decreased by 34% [45]. In epidemiological studies, there was a clear positive association between chronic mercury exposure and the risk of cardiovascular disease in people from European countries with low selenium levels, but no association was observed in a U.S. population with high selenium levels [46,47,48].

The present study employed a cross-sectional design; thus, causal inferences cannot be made. However, to minimize this limitation, we collected metabolic biomarker information at least six months after completing the collection of data regarding exposure and covariates. Moreover, considering that toenail mercury and selenium concentrations represent long-term exposure of up to one year [28,29,30,31,32], this decreases the likelihood of reverse direction bias. In addition, although we adjusted for known confounding variables in the models, some degree of residual confounding, possibly by factors that are unknown and cannot be measured, likely remained owing to the observational nature of the study. The mass of the toenail specimens and the season the nails were cut may affect trace element deposition in toenails, but we could not control for these factors in the analysis. Lastly, our study participants had higher blood mercury levels than those residing in other regions [41]; thus, the results may not be generalizable to residents in other regions if the biological mechanism of mercury, selenium, and MetS differs with variation in the mercury level.

## 5. Conclusions

Individuals who were older, had a higher BMI, smoked, had an alcohol drinking habit, had a higher household income, and consumed whale and shark meats were more likely to have higher toenail mercury levels than others were. Higher toenail mercury levels was associated with the risk of MetS, and this association was stronger in participants with a low toenail selenium concentration. To confirm the effects of methylmercury on metabolic health outcomes in an Asian population, considering modification in accordance with selenium exposure, a larger prospective cohort study or randomized controlled trial is needed.

## Figures and Tables

**Table 1:** Demographic and lifestyle characteristics of study participants.

Variables	*N* = 501
Age (years)	44.83 (±0.24)
Sex (%)	
Men	232 (46.3)
Women	269 (53.7)
BMI (%)	
Underweight	13 (2.6)
Normal	231 (46.2)
Overweight	138 (27.6)
Obesity	118 (23.6)
Smoking status (%)	
Non-smokers	327 (65.2)
Former smokers	80 (16.0)
Smokers	94 (18.8)
Alcohol consumption (%)	
Non-drinkers	107 (21.4)
Drinkers	394 (78.6)
Education (%)	
High school graduation or less	162 (32.4)
College graduation or more	338 (67.6)
Monthly household income (%)	
<3,000,000 won	108 (21.6)
3–3,990,000 won	122 (24.4)
4–4,990,000 won	89 (17.8)
5–5,990,000 won	76 (15.2)
≥6,000,000 won	106 (21.2)
Residence area (%)	
Urban	288 (57.5)
Rural	213 (42.5)
MET-h/week (%)	
<20	184 (37.7)
20–39	100 (20.5)
≥40	204 (41.8)
Family history of hypertension (%)	161 (32.3)
Family history of cardiovascular diseases (%)	52 (10.5)
Family history of diabetes (%)	110 (22.1)
Dietary supplement use (%)	284 (56.7)
Toenail mercury (µg/g)	0.40 (±0.01)
Toenail selenium (μg/g)	0.69 (±0.01)

Values are means ± standard error or *n* (%). BMI, body mass index; MET, metabolic equivalent.

**Table 2:** Dietary and non-dietary correlates of toenail mercury levels ^1,2^.

Variables	% Difference ^3^	*p* Value
Age (years)	1.2	0.005
Sex		
Men	reference	
Women	−6.0	0.4
BMI (kg/m^2^)		
Underweight	reference	
Normal	24.6	0.09
Overweight	30.5	0.04
Obesity	45.0	0.003
Smoking status		
Non-smokers	reference	
Former smokers	14.2	0.09
Smokers	23.1	0.005
Alcohol consumption		
Non-drinkers	reference	
Drinkers	14.8	0.01
Monthly household income (won)		
<3,000,000	reference	
3–3,990,000	11.1	0.1
4–4,990,000	7.0	0.3
5–5,990,000	16.8	0.03
≥6,000,000	18.2	0.009
MET-h/week		
<20	reference	
20–39	−2.0	0.7
≥40	−3.4	0.5
Residence area		
Urban	reference	
Rural	−0.9	0.8
Family history of cardiovascular diseases		
No	reference	
Yes	9.7	0.2
Shark and whale meat intake level (g/day)	14.8	<0.001
Total fish intake level (g/day) ^4^	0.1	0.2

^1^ Multivariable-adjusted including each variable in the table; ^2^ Toenail mercury level was logarithmically transformed as the dependent variable for the multiple linear regression analysis; ^3^ Beta-coefficient is the approximate % difference in toenail mercury (ln (μg/g)) from the reference category or per unit change in the variable; BMI, body mass index; MET, metabolic equivalent; ^4^ The sum of 21 fish and seafood items including: mackerel, anchovy, salmon, eel, tuna (fresh and canned), pollock/cod, yellow corvina/flounder, hair tail, rock fish/yellow tail/skate ray, file fish/monk fish/naked sand lance, puffer, sea bream, squid/octopus, shellfish, whelk/gastropods/urban/marsh snail, oyster/abalone, warty sea squirt, crab, shrimp, fish paste and seafood processing byproducts (salted seafood).

**Table 3:** Anthropometry and blood metabolic biomarkers according to the sex-specific tertiles of toenail mercury levels.

	Sex-Specific Tertile of Toenail Mercury (μg/g)						*p* for Trend
	First Tertile (*N* = 166)		Second Tertile (*N* = 168)		Third Tertile (*N* = 167)		
Body mass index (kg/m^2^)	22.7	± 0.2	23.0	± 0.2	24.0	± 0.2	<0.001
Waist circumference (cm)	75.8	± 0.8	78.0	± 0.8	79.0	± 0.7	<0.001
Systolic blood pressure (mmHg)	116.3	± 1.1	116.5	± 1.0	119.1	± 1.0	<0.001
Diastolic blood pressure (mmHg)	72.4	± 0.8	72.8	± 0.8	74.4	± 0.8	<0.001
Fasting blood glucose (mg/dL)	90.2	± 0.9	90.1	± 0.9	94.2	± 0.9	<0.001
Triglyceride (mg/dL)	107.0	± 5.3	110.8	± 5.3	119.8	± 5.3	<0.001
Total cholesterol (mg/dL)	191.2	± 6.8	207.3	± 6.7	191.6	± 6.8	0.7
HDL-cholesterol (mg/dL)	56.2	± 1.8	59.5	± 1.8	55.1	± 1.8	0.1
LDL-cholesterol (mg/dL)	113.3	± 2.2	117.9	± 2.2	116.0	± 2.2	0.2

Values are age-adjusted means ± standard error.

**Table 4:** Odds ratios and 95% confidence intervals for metabolic syndrome and its components according to the sex-specific tertiles of toenail mercury levels.

	Sex-Specific Tertile of Toenail Mercury (ln (μg/g))			*p* for Trend
	First Tertile	Second Tertile	Third Tertile	
Metabolic syndrome				
Case, *n*	9	7	19	
Model 1	1	0.78 (0.28−2.17)	2.29 (0.99−5.31)	0.06
Model 2	1	0.84 (0.29−2.44)	2.47 (1.01−6.08)	0.03

Data are presented as odds ratio (95% confidence interval); Model 1: adjusted for age; Model 2: additionally adjusted for monthly household income, smoking status, alcohol consumption, and physical activity level.

**Table 5:** Odds ratios (OR) and 95% confidence intervals (CI) for metabolic syndrome according to the sex-specific tertiles of toenail mercury, stratified by toenail selenium levels.

Metabolic Syndrome ^1^	Sex-Specific Tertile of Toenail Mercury (ln (μg/g))			*p* for Trend
	First Tertile	Second Tertile	Third Tertile	
Toenail selenium ≤0.685 μg/g				
Case, *n*	4	3	12	
Multivariate OR (95% CI) ^1^	1	0.78 (0.16−3.80)	3.97 (1.15−13.76)	0.02
Toenail selenium >0.685 μg/g				
Case, *n*	5	4	7	
Multivariate OR (95% CI) ^1^	1	0.95 (0.21−4.31)	1.56 (0.39−6.20)	0.5

^1^ Adjusted for age, monthly household income, smoking status, alcohol consumption, and physical activity level.
